# Anthraquinones and their analogues as potential biocontrol agents of rust and powdery mildew diseases of field crops

**DOI:** 10.1002/ps.6989

**Published:** 2022-06-10

**Authors:** Eleonora Barilli, Francisco J. Agudo, Marco Masi, Paola Nocera, Antonio Evidente, Diego Rubiales

**Affiliations:** ^1^ Institute for Sustainable Agriculture, CSIC Córdoba Spain; ^2^ Dipartimento di Scienze Chimiche, Università di Napoli Federico II Complesso Universitario Monte Sant'Angelo Naples Italy

**Keywords:** biocontrol, fungal metabolites, anthraquinones, pachybasin, powdery mildew, rust

## Abstract

**Background:**

Rusts and powdery mildews are severe fungal diseases of major crops worldwide, including cereals and legumes. They can be managed by chemical fungicide treatments, with negative consequences as environmental pollution and risk for human and animal health. Bioactive natural products could be the safest alternative for pest control. The family of anthraquinones, as well as analogue compounds containing an anthraquinone moiety or some modified anthraquinone rings, has been reported to exhibit certain antibiotic activity. Thus, the potential antifungal activity of some anthraquinones isolated from *Ascochyta lentis*, was assayed in this study for their effectiveness to reduce rust and powdery mildew diseases on pea and oat. Their effect on fungal development was macro‐ and microscopically assessed on inoculated leaves, and compared to the control achieved by the chemical fungicide (Tetraconazol 12.5% and Azoxystrobin 25%). In addition, the most promising compound was also tested at different concentrations in inoculated whole plants in order to evaluate its preventive and curative potential against fungal infection.

**Results:**

All metabolites studied strongly reduced the development of rust and powdery mildews in both pea and oat, being pachybasin and lentiquinone C the most effective ones in hampering fungal spore germination and appressoria formation. Some of them also affected post‐penetration events reducing colony size and number of haustoria per colony. Results were confirmed for pachybasin in whole plants assays, showing an efficacy similar to the commercial fungicide to control fungal diseases, both in preventive and curative applications.

**Conclusions:**

Some fungal anthraquinones and close metabolites, especially pachybasin, could be very promising molecules with effective potential as antifungal agents against both rust and powdery mildew of both pea and oat. Some structure activity‐relationships feature have also been evaluated. © 2022 The Authors. *Pest Management Science* published by John Wiley & Sons Ltd on behalf of Society of Chemical Industry.

## Graphical abstract

Biotrophic fungi as rust and powdery mildew can cause severe physiological damage in crops of agronomic importance. This article highlights the importance of some fungal anthraquinones and their analogues, especially pachybasin, which are very promising molecules with effective potential as safely biofungicide.

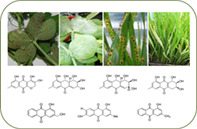

## 1 INTRODUCTION

Foliar diseases caused by biotrophic fungal pathogens, such as rusts and powdery mildews are major limiting factors for yield production in field crops worldwide, including legumes and cereals, and are responsible for losses ranging between 20–40% of global agricultural productivity.^1^ There are many species of rusts and powdery mildew fungi, being *Puccinia* and *Uromyces*, and *Blumeria* and *Erysiphe* the most important genus of rusts and powdery mildews, respectively.^2^, ^3^ Among these, *Puccinia coronata* f.sp. *avenae* and *Uromyces pisi* cause rust disease, and *Blumeria graminis* f.sp. *avenae* and *Erysiphe pisi*, cause powdery mildew diseases on oat and pea, respectively.^2^, ^4^

Biotrophic pathogens have very efficient spreading mechanisms, hampering their management. Several efforts were made to develop resistant cultivars,^1^, ^2^, ^4^ but to date, the use of chemical is the most diffuse method for rust and powdery mildew management,^5^, ^6^ even though fungicides have low specificity, are not easily biodegradable and pathogens tend to develop resistance after a prolonged use.^7^ These problems prompt the search for ecofriendly alternative methods also to satisfy the global pressing requests of policy makers and consumers.

Many efforts have been made to discover natural products with known and/or new structures and modes of actions. Among them bioactive natural products with potential and different practical applications in agriculture were recently isolated from different fungi,^8^, ^9^, ^10^ as well as from weeds and cultivated plants.^11^, ^12^ Cyclopaldic acid and *epi*‐epoformin^13^, ^14^ produced by *Seiridium cupressi* and *Diplodia quercivora*, pathogen of the forest plant cypress (*Cupressus sempervirens*) and oak (*Quercus canariensis*), respectively, have been reported to inhibit *Puccinia* and *Uromyces* spore germination and hypha infection.^15^, ^16^ Also cavoxin,^17^ inuloxin C^12^ and sphaeropsidin A^18^ have been reported to inhibit pea powdery mildew (*E. pisi*) spore germination and haustoria formation, resulting in disease reduction.^19^

Anthraquinones represent a well‐known class of natural compounds produced by plants and fungi.^20^, ^21^ They include phytotoxins produced by pathogenic fungi of field^22^ and forest crops.^23^ Among many examples, some different anthraquinones such as chrysophanol, emodin, pachybasin and ω‐hydroxypachybasin were isolated from *Trichoderma* spp.^24^ Others, such as lentiquinones A, B, and C and lentisone, which are close fungal metabolites, together with pachybasin, ω‐hydroxypachybasin and phomarin were recently isolated from *Aschochyta lentis*, the causal agent of lentil's Ascochyta blight. These anthraquinones have shown antibacterial, antiparasitic, insecticidal, fungicidal, and antiviral activities.^25^, ^26^, ^27^

On viewing these results, seven metabolites isolated from *A. lentis* (specifically lentiquinones A, B and C, lentisone, pachybasin, ω‐hydroxypachybasin and phomarin) belonging or closely related to the anthraquinone's family, were tested for their potential as natural fungicides against agronomically damaging pea and oat rusts (*Uromyces pisi* and *Puccinia coronata* f.sp. *avenae*) and pea and oat powdery mildews (*Erysiphe pisi* and *Blumeria graminis* f.sp. *avenae*) under controlled conditions both in detached leaves and in whole plant assays.

## 2 MATERIALS AND METHODS

### 2.1 Fungal bioactive metabolites

**Figure 1:**
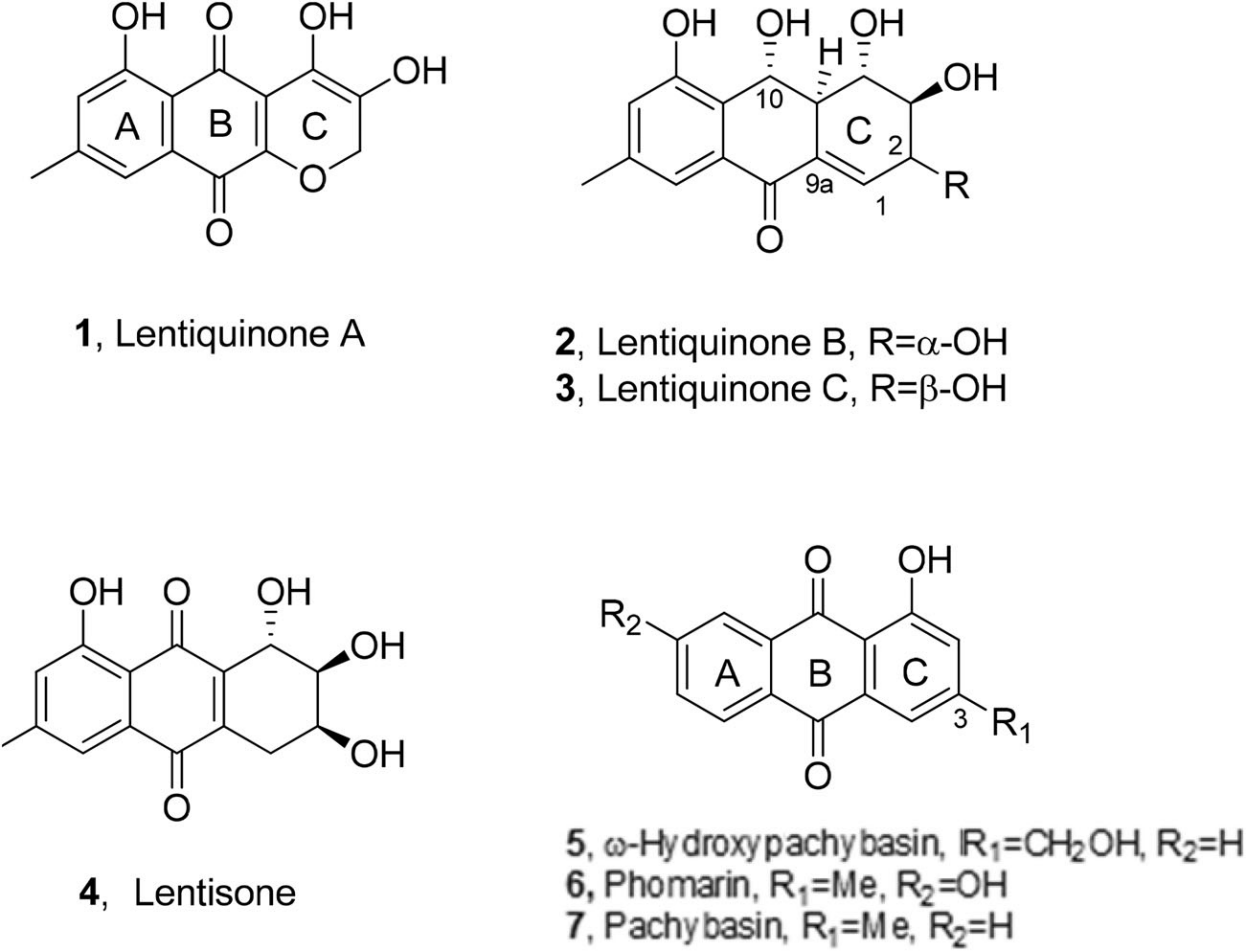
Structures of lentiquinones A–C, lentisone, ω‐hydroxypachybasin, phomarin and pachybasin (**1**–**7**).

The anthraquinones pachybasin, ω‐hydroxypachybasin and phomarin as well as the close lentiquinones A‐C, and lentisone (Fig. 1), were obtained from the culture filtrates and mycelium of *Ascochyta lentis* isolated from diseased lentil (*Lens culinaris*) plants and purified as recently detailed by Masi *et al*.^27^

### 2.2 Plant growth and pathogen multiplication

Seedlings of pea (*P. sativum* subsp. *sativum*) cv. Messire and oat (*Avena sativa*) cv. Selma, highly susceptible to pea and oat rusts and powdery mildews, respectively, were used both for pathogen multiplication and assays. Plants were raised from seeds in pots (6 × 6 × 10 cm) filled with a potting mixture (sand/peat, 1:3 vol/vol), then were grown in a growth chamber at 20 ± 2 °C and 65% relative humidity under a photoperiod at 14 h light/10 h dark with light intensity of 200 μmol m^−2^ s^−1^ photon flux density supplied by high‐output white fluorescent tubes.

**Figure 2:**
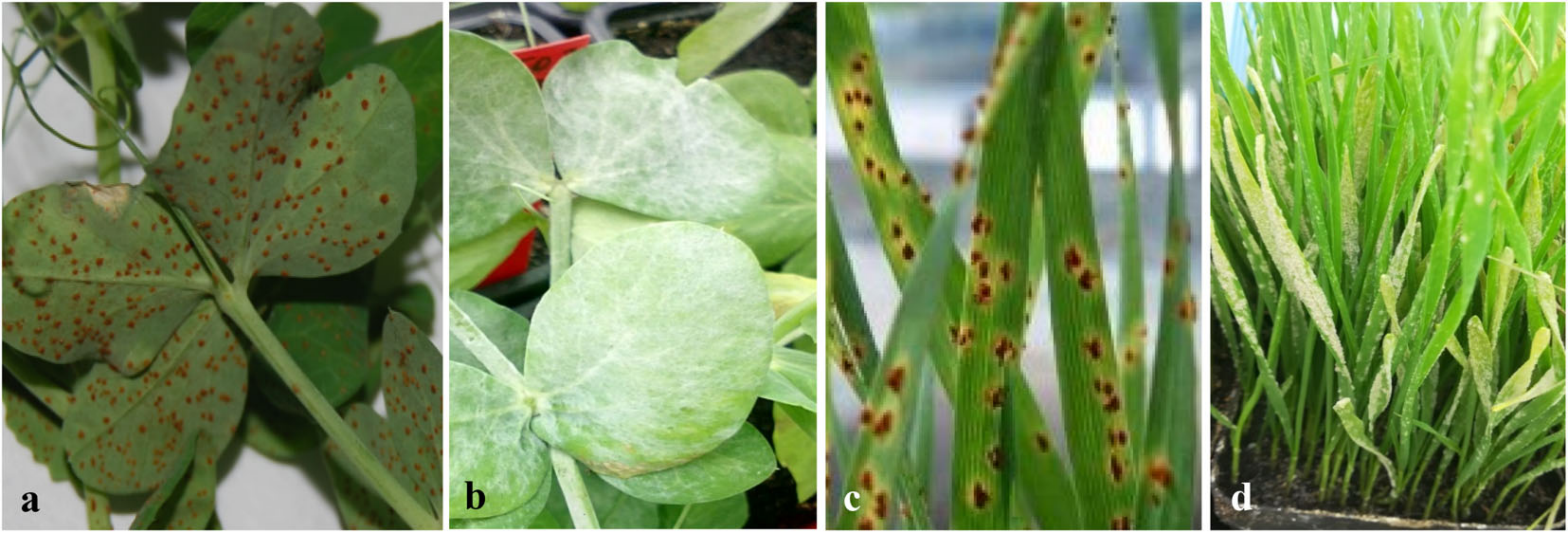
Susceptible pea (*Pisum sativum*) cv. Messire infected with *Uromyces pisi* (a) or with *Erysiphe pisi* (b); susceptible oat (*Avena sativa*) cv. Selma infected with *Puccinia coronata* f.sp. *avenae* (c) or with *Blumeria graminis* f.sp. *avenae* (d).

Both rust and powdery mildew are obligate biotrophic fungi, needing living plants for their multiplication as they do not grow in artificial culture media. Rust spores can easily be long term stored frozen or even short term maintained at air temperature. However, powdery mildew spores cannot be stored, and the fungus must be maintained on living plants. Because of this, the pea powdery mildew (*E. pisi*) isolate CO‐01 derived from mildew population collected at Córdoba (southern Spain) was maintained on Messire seedlings, and the oat powdery mildew (*Blumeria graminis* f.sp. *avenae*) race 5 was maintained on Selma seedlings. Both isolates are permanently maintained at IAS‐CSIC premises for use in different experiments. Leaves with heavy sporulation were shaken 1 day before inoculation ensuring that only young, vigorous spores were used for inoculum the following day (Fig. 2).

For rust, 12‐day‐old seedling plants of each species were inoculated with their respective fungal isolates, preserved at −80 °C, for spore multiplication. Rust isolates used were *U. pisi* isolate UPC‐04 and *P. coronata* f.sp. *avenae* isolate Co‐04 belonging to the IAS‐CSIC fungal collection. Plants were inoculated by dusting the leaves with rust urediospores (2 mg spores plant^−1^) mixed with pure talc (1:10, w:w) using a spore settling tower. Plants were incubated for 24 h at 20 °C in complete darkness and 100% relative humidity, and then returned to growth chamber conditions to allow disease development. Fresh urediospores were collected from leaves the same day of the bioassay. Every set of plants per isolate was maintained apart from the others in distinct growth chambers to avoid cross inoculations.

### 2.3 Fungal inoculations and disease assessments

#### 2.3.1 Detached leaves experiments

A first screening evaluating the macroscopical and microscopical effects of all metabolites was performed using a detached leaf method,^2^, ^28^ so that both inoculation density and incubation conditions could be more precisely controlled. Susceptible plants of pea cv. Messire and oat cv. Selma were grown under the controlled conditions mentioned above until the fifth leaf stage (pea) and the second‐formed leaf (oat) were achieved. Then several fourth‐formed (pea) and first leaves (oat) were excised and placed, adaxial side up, on 4% technical agar in Petri dishes. For each fungal pathogen, cut leaves were arranged in a randomized design with four replicates per treatment, each replicate having four leaves. The metabolites listed in Table 1 were tested at concentrations of 1 mM. Compounds were dissolved in MeOH (5%) and then brought up to the assay concentration with distilled water. The test solutions (50 μL) were applied on the adaxial leaf side together with negative controls consisting of distilled water and MeOH (5%) which were applied in the same amount (50 μL) as negative controls. Droplets (50 μL) of commercial anti‐powdery mildew fungicide (Tetraconazol 12.5%, Isagro® by Sipcan Inagra S.A.), or anti‐rust fungicide (Azoxystrobin 25%, Mirador® by Adama Agricultural Solutions Ltd.), were applied depending on the plant‐pathogen combination as positive control (0.2 gL^−1^). The solvent was evaporated in a laminar flow cabinet until dry. For each pathogen, the Petri dishes were inoculated in a spore settling tower spreading 50 fresh spores mm^−2^ collected from the susceptible inoculated genotypes (described in Section 2.2). Only for rust inoculation, plates were transferred during 24 h to a cabinet at 20 ± 2 °C in complete darkness and high relative humidity, and then were returned to growth chamber conditions. Twenty‐four hours after inoculation (h.a.i.), four leaves per plant, treatment and replicate were cut and used to perform microscopical studies on fungal development. Leaves were fixed by placing the adaxial surface upon filter paper moistened with 1:3 (v:v) glacial acetic acid: absolute ethanol. When the chlorophyll was eliminated, the leaves were transferred to filter paper moistened with tap water (minimum 2 h) to soften the tissue and then to filter paper moistened with lactoglycerol (1:1:1 v:v:v, lactic acid:glycerol:water). Fungal structures were staining with 0.25% Trypan blue, as described by Barilli *et al*.^29^ (for rust), or aniline blue in lactoglycerol (0.1%) (for powdery mildew)^28^ before examination by light microscopy using a phase contrast Leica DM LS microscope at ×20 and ×40 magnifications (Leica Microsystems, Wetzlar, Germany). On every leaf segment, germination frequency was assessed by scoring 100 spores for the presence of a germ tube. Further steps of fungal development were assessed on additional 35 randomly selected germinated spores, recording if appressoria was formed, and the number of hyphal tips and haustoria produced per colony. Finally, disease severity (DS%) was macroscopically assessed on an additional three inoculated cut leaves per repetition and treatment 2 weeks after inoculation by a visual estimation of the leaf area covered with rust pustules or with mildew mycelium. The presence or absence of necrosis indicating the phytotoxic effect of the metabolites tested was recorded.

**Table 1:** Physic data for anthraquinones, positive and negative control tested in pea and oat pathogens inhibition assays

Compounds	MW*
Lentiquinone A	274.2
Lentiquinone B	292.3
Lentiquinone C	292.3
Lentisone	290.3
ω‐Hydroxypachybasin	254.2
Phomarin	254.2
Untreated (control)	‐
Water (control)	‐
Water and MeOH 5% (control)	‐
Isagro® (Tetraconazol 12.5%)	‐
Mirador® (Azoxystrobin 25%)	‐

* Molecular weight.

A line has to be included in Table 1, between phomarin and untreated (control). In compounds coloumn you may put "pachybasin", and in MW column you should put 238.2.

#### 2.3.2 Whole‐plant assays

To simulate the effect of the treatments with metabolite against the different pathogens under real conditions, several experiments were carried out on whole plants of susceptible pea cv. Messire and oat cv. Selma, infected with either powdery mildew or rust under the controlled conditions mentioned in Section 2.2. Plants were arranged in a randomized design with five replicates per treatment, plant species and pathogen, each replicate having three plants. Two‐weeks old plants were inoculated by dusting the whole plants with fresh collected spores of rusts or powdery mildews. Each plant‐pathogen combination was inoculated apart, following procedures described in Section 2.3.1. Metabolite pachybasin, which was found to be the most effective in pea and oat detached leaves against both pathogens, was applied in whole plants at two concentrations as 1 and 2 mM. In order to study the protective effect of the metabolite, a set of plants was treated 2 days before fungal inoculation (DBI) and another set at the same time as fungal inoculation (0 DBI). In addition, to study the curative effect of the metabolites, a third set of plants was treated 2 days after fungal inoculation (DAI). Pachybasin was suspended in distilled water with MeOH (5%) and then sprayed over the canopy of the seedlings until run‐off. For the negative controls, plants were untreated (Control), sprayed with distilled water only (Water) or sprayed with distilled water with MeOH at 5%. The commercial fungicides for rust or powdery mildew depending on the assay, were applied as positive control at their recommended concentrations. Disease severity was assessed 7 or 10 days after inoculation, for powdery mildews and rusts respectively, as percentage of the whole plant covered by the symptoms. Attention was paid to observe eventual necrosis indicating the phytotoxic effect of the metabolites tested.

### 2.4 Statistics

For statistical analysis, percentage data were transformed to arcsine square roots (transformed value = 180/п × arcsine [√(%/100)]) to normalize data and stabilize variances throughout the data range. Transformed data were subjected to analysis of variance (ANOVA) using Statistix 8 (Analytical Software, Tallahassee, FL), after which residual plots were inspected to confirm data conformity to normality. Significance of differences between means was determined by calculating least significant difference (LSD) (*P* = 0.01).

## 3 RESULTS AND DISCUSSION

### 3.1 Detached leaves assays and microscopic assessments

In this study, four anthraquinones analogues (Fig. 1 **, 1**–**4**) and three anthraquinones (Fig. 1, **5**–**7**) previously isolated^27^ from the culture filtrates and mycelium of *A. lentis* were tested for their inhibitory effect on rusts and powdery mildews at all plant penetration stages, from spore germination till colony formation and sporulation. The metabolites studied were identified comparing their ^1^H NMR and ESI MS spectra and specific optical rotation with those previously reported.^27^ Their purity was higher than 95% and was ascertained by ^1^H NMR and HPLC analyses.

#### 3.1.1 Plant‐rust interactions

For rusts, when environmental conditions are favorable, urediospores germinate forming a germ tube that grows over the leaf surface until a stoma is recognized. Then, an appressorium is differentiated over a stoma and a substomatal vesicle is formed inside the stomatal cavity from which the fungus grows intercellularly towards the mesophyll forming infection hyphae that, after coming into contact with the mesophyll cells wall, originates the haustorial mother cells. This structure originates a nutrient‐absorbing haustorium and, from this moment the pathogen's development is supported by host metabolism.^30^ Already by 24 h after inoculation (h.a.i.) the two rust fungi studied (*Uromyces pisi* on pea and *Puccinia coronata* f.sp. *avenae* on oat) showed high percentage of spore germination (>70%) in the untreated and negative controls, with no significant differences among them. By contrary, spore germination was markedly reduced (<2.4%) by the fungicide (Azoxystrobin 25%) (Table 2), as expected since its efficacy in reducing rust germination and subsequent fungal penetration stages is well known in several crops including legumes and cereals.^5^, ^31^ Also lentiquinone C and pachybasin markedly inhibited spore germination of both rusts at similar levels than the commercial fungicide (range 1.9–6.4%) (Table 2). Remaining metabolites also significantly inhibited spore germination of rusts as compared with the untreated control (in the range of 1.5–3.4 and 2.5–3.5 fold reduction in pea and oat, respectively), but not as strongly as lentiquinone C and pachybasin (Table 2).

**Table 2:** Microscopical observations on the effect achieved by seven metabolites (at 1 mM) on rusts development stages at 24 hai*

	Pea – *Uromyces pisi*				Oat – *Puccinia coronata* f.sp. *avenae*			
Treatments	Microscopical data				Microscopical data			
	% Germination	% Appressoria	No of hypal tips/colony	No of haustoria/colony	% Germination	% Appressoria	No of hypal tips/colony	No of haustoria/colony
Untreated	79.1 ± 4.3 a	54.9 ± 1.8 a	3.6 ± 1.1 a	1.8 ± 0.7 a	79.4 ± 3.7 a	53.6 ± 0.6 a	3.5 ± 0.6 a	1.9 ± 0.5 a
Water control	78.9 ± 3.7 a	52.5 ± 2.1 a	3.5 ± 1.1 a	1.8 ± 0.5 a	79.0 ± 3.8 a	52.8 ± 1.4 a	3.3 ± 0.5 a	1.7 ± 0.2 a
MeOH 5% control	77.1 ± 3.4 a	52.7 ± 2.8 a	3.2 ± 1 ab	1.7 ± 0.8 a	78.0 ± 4.3 a	53.4 ± 1.2 a	3.4 ± 0.9 a	1.8 ± 0.9 a
Lentiquinone A 1 mM	39.3 ± 0.5 b	16.7 ± 1.5 b	2.9 ± 0.8 bc	1.6 ± 0.9 ab	28.4 ± 3.1 b	11.8 ± 0.5 b	2.6 ± 0.6 ab	1.3 ± 0.5 ab
Lentiquinone B 1 mM	52.0 ± 4.3 ab	19.4 ± 1.2 b	3.1 ± 1 ab	1.3 ± 0.4 bc	31.2 ± 5.9 b	2.5 ± 0.3 d	2.6 ± 0.4 ab	1.4 ± 0.4 ab
Lentiquinone C 1 mM	6.4 ± 2.1 bc	2.7 ± 0.9 de	1.2 ± 0.5 d	0.5 ± 0.4 d	1.9 ± 1.1 d	0.1 ± 0.03 fg	0.8 ± 0.2 b	0.3 ± 0.15c
Lentisone 1 mM	23.2 ± 3.6 b	14.8 ± 0.8 bc	3.5 ± 0.6 a	1.9 ± 0.8 a	28.7 ± 2.9 b	2.3 ± 0.3 d	3.2 ± 0.7 a	1.6 ± 0.5 a
ω‐Hydroxypachybasin 1 mM	35.5 ± 2.7 b	14.0 ± 1.0 bc	2.2 ± 0.4 c	1.2 ± 0.6 c	29.2 ± 2.8 b	7.0 ± 0.9 c	1.4 ± 0.4 b	1.2 ± 0.2 b
Phomarin 1 mM	46.0 ± 1.6 b	13.6 ± 0.7 c	2.9 ± 1.0 bc	1.3 ± 0.5 bc	22.4 ± 1.0 b	1.2 ± 0.3 e	3.0 ± 0.4 a	1.4 ± 0.6 ab
Pachybasin 1 mM	3.4 ± 1.1 c	0.4 ± 0.5 e	0 ± 0 e	0 ± 0 e	2.7 ± 0.2 e	0.3 ± 0.2 fg	0 ± 0 c	0 ± 0 d
Fungicide Azoxystrobin 25%	1.5 ± 0.4 d	0.4 ± 0.4 e	0 ± 0 e	0 ± 0 e	0.6 ± 0.2 e	0 ± 0 g	0 ± 0 c	0 ± 0 d

Experiments were performed on susceptible pea and oat genotypes.

Values, per column and treatment, followed by different letters differ significantly at *P* < 0.01.

* Hours after inoculation.

**Figure 3:**
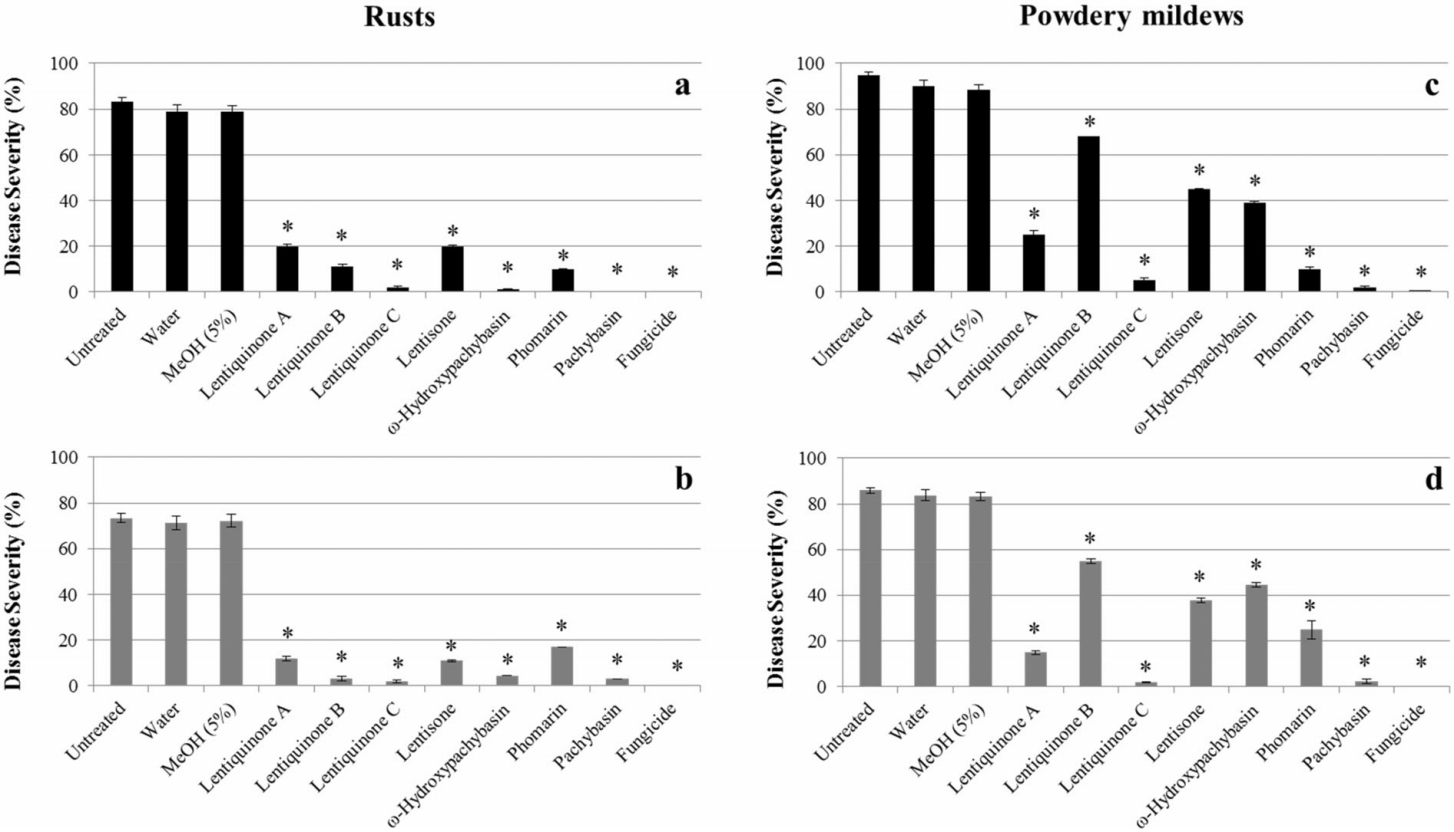
Disease severity (%) of pea and oat rusts (black and grey columns, respectively) (a) and pea and oat powdery mildews (black and grey columns, respectively) (b) in cut leave assays on negative (untreated, water and MeOH 5%) controls, treated leaves with metabolites and positive controls (fungicide). The compounds were tested at a concentration of 1 mM. The experiment was repeated four times. For each metabolite, the asterisk indicates that differences on fungal development due to the metabolite tested compared to the negative controls were significant (*P* < 0.01).

Germtube elongation and successful appressoria formation of germinated spores was not affected by the negative controls but were almost suppressed (<1% appressoria formation) by the commercial fungicide (Table 2), and significantly inhibited at various levels by all anthraquinones and close metabolites studied (range 0.1–19.4%). All the compounds tested reduced *U. pisi* and *P. coronata* f.sp. *avenae* appressorial formation below 20% and 11%, respectively (Table 2, Fig. 3). In particular, lentiquinone C and pachybasin were very active in reducing appressoria formation in both rusts, showing a fungitoxic effect that was comparable to those obtained with the chemical at the same concentration (<2.7%) (Table 2). Microscopically, as observed for spore germination, a high number of hyphal tips per colony (values up to 3) and haustoria per colony (range between 1.3 and 2) were achieved by the fungus in the negative controls (untreated, water and MeOH 5%), with no significant differences between them (Table 2). On the contrary, no colony development was observed in inoculated leaves treated with the commercial fungicide (Table 2). Lentiquinone C strongly inhibited both the number of hyphal tips and haustoria per colony in both rust pathosystems (hyphal tips per colony <1.2 and haustoria per colony <0.2), while pachybasin completely inhibited fungal expansion with no hyphal growth tips and no haustoria at all. These results were comparable with those obtained with the chemical at the same concentration. This is the first report on the inhibition effect exerted by antraquinones and close metabolites as pachybasin and lentiquinone C on rust pathogens, on early fungal development steps. This is in line with macroscopical observations on DS (% of pustules covering leaf tissue) which was about 80% in pea and 70% in oat negative controls, but underwent a strong reduction in all metabolites treated leaves (DS < 20% (Fig. 3), particularly high for lentiquinone C and pachybasin (<4%), similar to the commercial fungicide.

We did not observe any sign of phytotoxicity caused by any of the metabolites used for this study on plant hosts (both pea and oat) at the concentration tested. This is in line with results found by Masi *et al*.^27^ which highlighted the relation between the quinone skeleton of several anthraquinone analogues and their phytotoxicity.

Considering the results obtained, some structure–activity relationships could be hypothesized. Among the anthraquinone analogues (lentiquinones A–C and lentisone) the significant activity of lentiquinone C could be attributed to the different stereochemistry of the hydroxy group at C‐2 comparing to the structure of lentiquinone B. However, the reduction of the ketone group at C‐10 and the presence of a double bond between C‐1 and C‐9a also seem to be important for the activity considering that lentisone has the same stereochemistry of lentiquinone C for the carbons of C‐ring but it does not have these substitutions. The lower activity of lentiquinone A could be explained for the presence of a pyran C‐ring, which is not present in the other three analogues. Among the anthraquinones (pachybasin, ω‐hydroxypachybasin and phomarin), the strong activity of pachybasin could be due to the presence of a methyl group at C‐3 of the C‐ring and the simultaneous absence of substituents at A‐ring of the anthraquinone system which could differently affect their oxidoreductive ability.^21^, ^27^

#### 3.1.2 Plant‐powdery mildew interactions

The biological cycle of powdery mildews includes the germination of conidia on the host epidermis over which, as a difference with rusts, the pathogen grows epiphytically, invaginating haustoria into epidermal cells for feeding, but then forming secondary mycelia followed by subsequent epiphytic colony growth.^32^

On pea‐ and oat‐powdery mildew interactions studied on detached leaves, the percentage of fungal spore germination was higher than 70% and 60%, respectively, in all the negative controls (untreated, water and MeOH 5%), with no significant differences between them (Table 3). On the contrary, the commercial fungicide (Tetraconazol 12.5%) strongly reduced spore germination (<2%) showing its well‐known activity on powdery mildew development.^33^ Pachybasin was particularly effective reducing spore germination of both powdery mildew species (<6%). This was followed by lentiquinone C that was also very effective reducing oat powdery mildew spore germination (8.9%), although not that much for the pea pathogen (39.3%). All other metabolites also significantly reduced germination but this reduction was more modest (in the range 31.8–46.7%). There were no signs of phytotoxicity on leaves.

**Table 3:** Microscopical observations on the effect achieved by seven metabolites (at 1 mM) on powdery mildew development stages at 24 hai*

	Pea – *Erysiphe pisi*				Oat – *Blumeria graminis* f. sp. *avenae*			
Treatments	Microscopical data				Microscopical data			
	% Germination	% Appressoria	No of hypal tips/colony	No of haustoria/colony	% Germination	% Appressoria	No of hypal tips/colony	No of haustoria/colony
Untreated	77.6 ± 5 a	52.1 ± 3.7 a	11.2 ± 3.8 a	4.8 ± 1.8 a	72.8 ± 5.3 a	45.4 ± 4.3 a	6.9 ± 1.9 a	3.4 ± 0.9 a
Water control	75.8 ± 3.6 a	51.2 ± 4.3 a	10.4 ± 2.9 a	4.5 ± 1.3 a	70.2 ± 4.6 a	44.9 ± 4.6 a	6.3 ± 1.7 a	3.1 ± 0.7 a
MeOH 5% control	75.5 ± 3.7 a	51.7 ± 3.6 a	10.4 ± 2.6 a	4.3 ± 1.6 a	69.2 ± 4.7 a	44.3 ± 4.3 a	6.4 ± 1.4 a	3.1 ± 0.7 a
Lentiquinone A 1 mM	40.6 ± 2.9 bc	45.1 ± 2.9 b	2.3 ± 2.0 c	1.6 ± 0.9 b	43.0 ± 6.8 b	44.9 ± 4.1 a	3.8 ± 1.5 b	1.2 ± 0.8 bc
Lentiquinone B 1 mM	41.0 ± 5.0 bc	44.9 ± 3.9 b	3.7 ± 1.4 b	1.4 ± 0.8 b	31.8 ± 3.6 c	33.1 ± 2.9 b	3.1 ± 0.4 bc	1.5 ± 0.6 b
Lentiquinone C 1 mM	38.3 ± 1.7 c	5.8 ± 2.9 e	1.8 ± 1.2 c	0.4 ± 0.3 c	8.9 ± 3.1 d	11.0 ± 1.6 de	3.8 ± 1.3 b	0.7 ± 0.4 c
Lentisone 1 mM	46.7 ± 2.6 b	16.9 ± 4.1 d	4.4 ± 1.6 b	2.2 ± 0.8 ab	45.2 ± 2.8 b	23 ± 4.8 c	3.6 ± 1.2 b	1.7 ± 0.6 b
ω‐Hydroxypachybasin 1 mM	45.9 ± 2.9 b	46.9 ± 2.9 b	3.7 ± 1.5 b	0.6 ± 0.4 bc	38.8 ± 2.4 bc	31.4 ± 3.0 b	2.2 ± 0.8 c	0.5 ± 0.4 cd
Phomarin 1 mM	40.8 ± 4.9 bc	33.7 ± 2.6 c	3.1 ± 1.4 bc	1.4 ± 0.7 b	40.9 ± 4.2 bc	31.1 ± 5.5 b	3.6 ± 1.0 b	1.5 ± 0.6 b
Pachybasin 1 mM	5.8 ± 1.6 d	7.05 ± 1.9 e	1.4 ± 0.7 cd	0.3 ± 0.2 c	4.6 ± 2 d	7.5 ± 2.1 e	1.7 ± 0.8 cd	0.3 ± 0.2 d
Fungicide Tetraconazol 12.5%	1.6 ± 0.8 e	0.5 ± 0.2 f	1.3 ± 0.6 d	0.3 ± 0.2 c	0.9 ± 0.3 e	0.8 ± 0.5f	1.4 ± 0.7 d	0.25 ± 0.2 d

Experiments were performed on susceptible pea and oat genotypes.

Values, per column and treatment, followed by different letters differ significantly at *P* < 0.01.

* Hours after inoculation.

The successive appressoria formation stage of both powdery mildews was also strongly reduced by lentiquinone C (<11%) and pachybasin (<7.4%) compared to their controls. These compounds also strongly inhibited colony growth (<1.7 hyphal tips, with <1 haustoria per colony) in both pathosystems (Table 3). No associated phytotoxicity was observed for any treatment or pathosystem, in agreement to what found in Section 3.1.1.

Our results show that pachybasin and lentiquinone C strongly inhibit both pre‐ and post‐penetration stages of rusts and powdery mildews. Uredospores and conidia respond to host leaf treatment with a failure in germination and, successively, with a decrease in the proportion of normally developed appressorium competent for host penetration, which lead to a reduced and smaller final number of fungal colonies produced. These quantitative *in vitro* data suggest that this specific morphological response is as consequence of the direct dose‐dependent contact with the compounds. This is in accordance with previous results found for pachybasin tested *in vitro* against *B. graminis* f.sp. *hordei* (the causal agent of powdery mildew in barley) at different concentrations ranging between 10^−4^ and 1 mM,^34^ and showing a dose‐dependent efficacy in inhibit conidia germination and subsequent appressoria formation. In fact, Hildebrandt *et al*. showed that pachybasin only significantly affected the fungal pre‐penetration processes at the higher concentration tested, what is in agreement with our observations. Lentiquinone C has previously showed antibiotic and herbicide effects,^27^ but this is the first report on its fungicide capacity.

### 3.2 Studies performed *in planta*

Pachybasin, followed by lentiquinone C appeared the most effective anthraquinones inhibiting rusts and powdery mildews, when applied at a concentration comparable with the commercial fungicide. Pachybasin was produced in larger amounts by the fungus and thus was selected for further validations on whole‐plant experiments. While the applied metabolites in *in vitro* system facilitates a more or less homogeneous and reproducible conditions with lipophilic compounds, their efficient deposition by leaf spraying is highly dependent on the properties of the plant cuticle, the efficacy of spray mixture adjuvants, as well as by the environmental conditions, which clearly affects the general comparability of *in vitro* and *in vivo* data sets. Pachybasin was sprayed on whole plants at two concentrations (1 and 2 mM), before and after fungal inoculation to test its preventive and curative effects.

**Figure 4:**
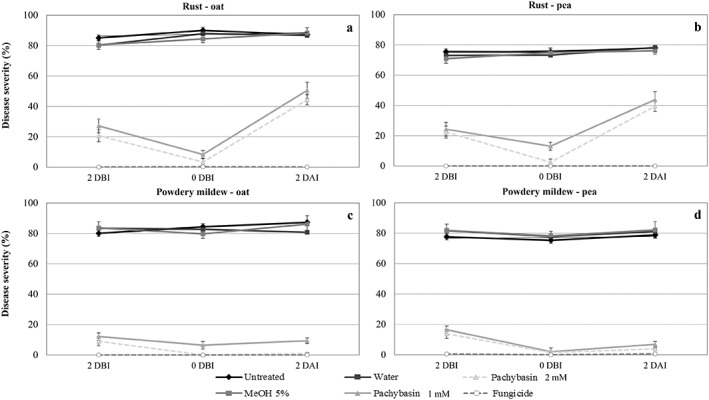
Disease severity (%) measured on rust‐inoculated whole plants of oat (a) and pea (b) at 10 days after inoculation, and on powdery mildew‐inoculated whole plants of oat (c) and pea (d) at 7 days after inoculation. Plants were treated at different time points (2 days before inoculation (2 DBI), the same day of fungal inoculation (0 DBI) and 2 days after inoculation (2 DAI)) with pachybasin at two different concentrations (1 and 2 mM), and were compared with positive controls (fungicides) and negative controls (untreated, water and MeOH 5%).

In the experiments with rusts, high IT values (=4), indicating a fully compatible interaction not associated with host cell necrosis at the infection site, were observed on both the pea and the oat accession in all treatments, showing that the metabolites did not induce hypersensitive resistance (Table 4). Treatments with pachybasin provided a significant reduction of DS for both rusts (<50%) compared with the negative controls (DS > 70%) (Table 4). Pachybasin was particularly effective when applied at the time of inoculation (0 DBI, <8% DS), followed by protective application 2 days earlier (2DBI, <27% DS), being less effective although still significant when applied after fungal inoculation (2 DAI, <51% DS) (Fig. 4(a),(b)). The concentration of the metabolite made little difference, being the lowest dose tested equally significant than the commercial fungicide in reducing the disease. The significant curative effect exhibit by pachybasin is in agreement with the inhibitory effects on early stages of fungal infection, from spore germination to early haustoria formation that normally occurs within the first 24 h, so these effects are not seen when treating 2 DBI. However, the fact that there is still a significant DS reduction by about half, confirms a curative effect reducing further growth of already formed fungal colonies. No symptoms of phytotoxicity were already observed, confirming previous observations.

**Table 4:** Macroscopical observations: infection type (IT) and final disease severity (DS%) of rust on plant of oat cv. Selma inoculated with *Puccinia coronata* f.sp. *avenae* and of pea cv. Messire inoculated with *Uromyces pisi* mM

	Oat – *P. coronata* f.sp. *avenae*						Pea – *U. pisi*					
Treatments	2 DBI		0 DBI		2 DAI		2 DBI		0 DBI		2 DAI	
	IT	DS%	IT	DS%	IT	DS%	IT	DS%	IT	DS%	IT	DS%
Untreated	4	85.0 ± 2.0 a	4	90.0 ± 2.0 a	4	87.2 ± 1.6 a	4	75.5 ± 1.5 a	4	75.3 ± 1.2 a	4	78.0 ± 1.4 a
Water control	4	80.3 ± 3.2 a	4	87.8 ± 2.3 a	4	86.8 ± 3.1 a	4	73.0 ± 1.3 a	4	73.2 ± 1.2 a	4	78.3 ± 1.1 a
MeOH 5% control	4	80.4 ± 3.0 a	4	84.4 ± 3.1 a	4	88.3 ± 3.4 a	4	70.9 ± 1.2 a	4	74.9 ± 1.2 a	4	76.1 ± 1.9 a
Pachybasin 1 mM	4	27.2 ± 4.6 b	4	8.3 ± 2.6 b	4	50.5 ± 5.4 b	4	24.3 ± 2.0 b	4	13.1 ± 1.7 b	4	43.8 ± 1.3 b
Pachybasin 2 mM	4	20.7 ± 3.9 b	4	3.4 ± 2.1 c	4	44.4 ± 3.4 b	4	22.4 ± 1.7 c	4	2.5 ± 0.8 c	4	39.4 ± 1.6 c
Fungicide Azoxystrobin 25%	0	0 ± 0 c	0	0 ± 0 d	0	0 ± 0 c	0	0 ± 0 c	0	0 ± 0 d	0	0 ± 0 c

DS% was assessed on whole planta 10 DAI.* Pachybasin selected metabolite, negative (untreated, water and MeOH 5%) and positive (Azoxystrobin 25% fungicide) controls were applied to plants at different times to fungal inoculation: 2 DBI,^†^ 0 DBI and 2 DAI. Pachybasin was tested at a concentration of 1 and 2.

Values, per column and treatment, followed by different letters differ significantly at *P* < 0.01.

* Days after inoculation.

^†^ Days before inoculation.

Similarly, treatments with pachybasin provided a significant control of both powdery mildews (DS% < 17) compared with the negative controls (DS% > 75) (Table 5; Fig. 4(c),(d)). Differently than with rusts, pachybasin showed similarly good curative and protective effects (DS < 17% at 2DBI, <7% at 0DBI; <10% at 2 DAI). This can be explained by the ectotrophic growth of powdery mildew, which allowed that the metabolite enters into direct contact with fungal mycelia even when applied later. On the contrary rusts grow over the epidermis only until the stage of appressoria formation, with all subsequent fungal growth occurring inside substomatal vesicle or the mesophyll.

**Table 5:** Macroscopical observations: infection type (IT) and final disease severity (DS%) of powdery mildew on plants of pea cv. Messire and oat cv. Selma inoculated with *Erysiphe pisi* and *Blumeria graminis* f.sp. *avenae*, respectively

	Oat – *B. graminis* f.sp. *avenae*						Pea – *E. pisi*					
Treatments	2 DBI		0 DBI		2 DAI		2 DBI		0 DBI		2 DAI	
	IT	DS%	IT	DS%	IT	DS%	IT	DS%	IT	DS%	IT	DS%
Untreated	4	80.0 ± 2.2 a	4	84.3 ± 2.9 a	4	87.2 ± 1.28 a	4	77.6 a ± 3.5 a	4	75.3 ± 2.8 a	4	78.8 ± 2.4 a
Water control	4	83.5 ± 4.0 a	4	82.7 ± 2.7 a	4	80.7 ± 5.8 a	4	81.7 ± 2.1 a	4	77.8 ± 1.9 a	4	81.0 ± 1.5 a
MeOH 5% control	4	83.6 ± 4.2 a	4	79.7 ± 3.1 a	4	86.1 ± 5.5 a	4	81.8 ± 2.4 a	4	78.3 ± 1.5 a	4	82.2 ± 1.5 a
Pachybasin 1 mM	4	12.1 ± 2.5 b	4	6.6 ± 6.5 b	4	9.4 ± 1.8 b	4	16.5 ± 2.8 b	4	2.0 ± 0.9 b	4	6.9 ± 2.8 b
Pachybasin 2 mM	4	9.0 ± 3.0 b	4	0 ± 0 c	4	1.0 ± 0.6 c	4	13.8 ± 2.6 b	4	1.4 ± 1.0 bc	4	3.9 ± 1.8 b
Fungicide Tetraconazol 12.5%	0	0 ± 0 c	0	0 ± 0 c	0	0 ± 0 c	0	0.5 ± 0.1 c	0	0 ± 0 c	0	0.6 ± 0.2 c

DS% was recorded on whole plants 7 DAI.* Pachybasin selected metabolite, negative (untreated, water and MeOH 5%) and positive (Tetraconazole 12.5% fungicide) controls were applied to plants at different times to fungal inoculation: 2 DBI,^†^ 0 DBI and 2 DAI. Pachybasin was tested at a concentration of 1 and 2 mM.

Values, per column and treatment, followed by different letters differ significantly at *P* < 0.01.

* Days after inoculation.

^†^ Days before inoculation.

Due to the strong fungal inhibition of pachybasin, we could suppose that repeated preventive application of this anthraquinones may effectively reduce the major part of the fungal infection potential.

## 4 CONCLUSIONS

Biotrophic fungi as powdery mildew and rust fungi are among the most common and important plant fungal pathogens of both cereal and legume crops. Although cultural and biological practices contribute to reduce the risk of disease development, they do not provide sufficient protection. As a result, chemical control including the use of commercial fungicide from multiple chemical groups is, in practice, the most effective tool for managing these pathogens when no genetic resistance is available. Unfortunately, the risk of resistance development is high because typical protective programs include multiple applications per season. In addition, some of the most economically destructive species of biotrophic fungi are considered to be high‐risk pathogens and are able to develop resistance to several chemical classes within a few years. This situation has decreased the efficacy of the major fungicide classes that are employed against both rusts and powdery mildews leading to the application of general integrated disease management strategies as dose limitation, mixtures and search of alternative molecules.

With respect to this last point, many plant pathogens, especially necrotrophic fungi, are capable of producing a broad panel of natural substances representing an unexploited source of potential biofungicides with new molecular structures and mode of actions against several crop diseases. Nevertheless, the choice of the best metabolite as well as the optimal dose and time of application varied greatly depending on the pathosystem involved. In fact, for biotrophic plant fungi of agronomic importance as rusts and powdery mildews, a successful initial spore germination and fungal penetration are crucial phases for disease development. Here, seven fungal metabolites originate from *A. lentis* and belonging to the anthraquinone‐analogues natural compounds were tested with a general success, for their fungitoxic activity against several powdery mildew and rusts of agronomic importance. Lentiquinone C and pachybasin were both promising compounds reducing *in vitro* the early developmental stages of *U. pisi*, *P. coronata* f.sp. *avenae*, *E. pisi* and *B. graminis* f.sp. *avenae*, as well as their post‐penetration development at values comparable to those obtained by chemical protection. Large amount of pachybasin is produced by *A. lentis* mycelium (7 g kg^−1^) while only 0.4 mg L^−1^ of lentiquinone C could be obtained from its culture filtrate.^27^ For this reason the former compound was the most interesting for *in planta* studies, where inhibition of fungal growth at pre‐ and post‐penetration stages, with consequent interesting preventive and curative effects against fungal infection, were exhibited. Due to the pronounced similarities with respect the limitation in germ tube formation and following steps of pathogen development between all biotrophic fungi tested here, it is tempting to hypothesize that pachybasin might exert their antifungal activity with a direct antifungal activity and/or also by modifying in a certain manner the host perception from the pathogen. In fact, the germ tubes of biotrophic fungi possess high capability for sensing host topographical signals^35^ and to recognize host‐derived volatiles as signals to stimulate and regulate certain following steps of fungal development process. It has been demonstrated that other molecules such as farnesyl acetate acted as an antagonistic signal and strongly regulated the rate of appressoria differentiation and colony development in several rust species.^36^ Additional studies in this direction may be of interest to better understand the biological properties of pachybasin, which will lead us to an adequate use of this compound in agriculture against biotrophic pathogens of agronomic interest.

In addition, further ecotoxicological studies will be performed in the near future as required to realize the scale‐up of pachybasin production in big fermenters and its bioformulation is feasible in order to make the compound safe and economical for its use in agriculture.

## Data Availability

The data that support the findings of this study are available on request from the corresponding author. The data are not publicly available due to privacy or ethical restrictions.
